# Continuous blood purification treatment for endotoxin-induced acute respiratory distress syndrome

**DOI:** 10.1590/1414-431X20165367

**Published:** 2017-02-16

**Authors:** Y. Jiang, R. Lin, Y. Xu, S. Zhang, K. Cui, M. Zhu, A. Li, C. Chen, J. Yang, W. Yang

**Affiliations:** 1 Intensive Care Unit, Taizhou Hospital of Zhejiang Province, Wenzhou Medical University, Taizhou, China; 2 Medical Research Center, Taizhou Hospital of Zhejiang Province, Wenzhou Medical University, Taizhou, China

**Keywords:** Acute respiratory distress syndrome, Continuous blood purification treatment, Endotoxin, Hemodynamics

## Abstract

This study aimed to explore the effects of continuous blood purification (CBP) treatment in pigs affected with acute respiratory distress syndrome (ARDS). A total of 12 healthy male pigs, weighing 12±1.8 kg, were randomly and equally assigned to the control and experimental groups. The ARDS pig model was prepared by intravenous injections of endotoxin (20 µg/kg). The control group was given conventional supportive therapy, while the experimental group was given continuous veno-venous hemofiltration therapy. During the treatment process, the variations in dynamic lung compliance, oxygenation index, hemodynamics, and urine volume per hour at different times (Baseline, 0, 2, 4, and 6 h) were recorded. The levels of tumor necrosis factor (TNF-α), interleukin 6 (IL-6), and IL-10 in serum and bronchoalveolar lavage fluid (BALF) were measured using the enzyme-linked immunosorbent assay. The histomorphological changes of the lung, heart, and kidney were visualized using a light microscope. The nuclear factor κB p65 protein content of the heart, lung, and kidney tissues was also detected using western blot. The experimental group outperformed the control group in both respiratory and hemodynamic events. CBP treatment cleared TNF-α, IL-6, and IL-10 partially from serum and BALF. The pathological examination of the heart, lung, and kidney tissues revealed that the injury was less severe in the experimental group. CBP treatment can improve the organ functions of pigs affected with endotoxin-induced ARDS and protect these organs to some extent.

## Introduction

Acute respiratory distress syndrome (ARDS) is a catastrophic syndrome commonly seen among intensive care unit patients, with a fatality rate of 27–45% (1). Its main features are pulmonary edema and refractory hypoxemia caused by infiltration of numerous neutrophils, inflammation in lung tissues, and damage of alveolar capillary barriers (2,3). Currently, ARDS has no effective pharmacological treatment; therefore, it is mainly managed symptomatically, and lung-protective ventilation strategies with high positive end-expiratory pressure and low tidal volume are widely accepted and applied (4). Overwhelming inflammation resulting from insufficient recession or excessive release of proinflammatory mediums is one of the main factors in the pathogenesis of ARDS. To improve the prognosis of patients, the lung tissues should be protected by reducing the lung water content and inflammation reactions (1).

Continuous blood purification (CBP) treatment has been widely used in renal replacement therapy of patients with severe syndrome. As this treatment can non-selectively remove inflammatory mediators and stabilize hemodynamics, it has also become popular in recent years in treating sepsis, multiorgan dysfunction, congestive heart failure, and other diseases (5–7). ARDS is the result of overwhelming pulmonary inflammation and has also been shown to induce multiorgan dysfunction. Some studies have proven that CBP can improve the prognosis of patients with ARDS, although it is not the standard treatment of ARDS. Whether CBP can be adopted for treating ARDS is still controversial; therefore, this study aimed to evaluate the effect of CBP treatment in organ protection of pigs affected with endotoxin (lipopolysaccharide)-induced ARDS.

## Material and Methods

### Grouping and processing

*Animal modeling*. A total of 12 healthy male piglets (12±1.8 kg) were provided by Taizhou Taihe Biological Technology Co. Ltd., China. The experiments performed in this study were approved by the Animal Ethics Committee of the Taizhou Hospital of Zhejiang. The piglets were fasted for 8 h prior to the experiment. The piglets were anesthetized with intramuscular midazolam (3 mg/kg), and then a computed tomography (CT) scan of the lungs was performed. The left internal jugular vein was opened for fluid infusion, and the right femoral artery was connected to the pulse contour cardiac output (PICCO) monitor (Pulsion, Germany). The right femoral vein was catheterized with a pediatric blood filter pipeline for CBP. The bladder was then punctured under the guidance of ultrasound positioning, and the urine was drained. A combination of fentanyl citrate and propofol was used for procedural sedation and anesthesia. Endotracheal intubation was then equipped, and the pipe was connected to a breathing machine for auxiliary ventilation (Newport e360, Newport Medical Instruments, USA). For the respiratory pattern, the synchronized intermittent mandatory ventilation plus pressure support ventilation mode was chosen, which followed the small-tidal-volume lung-protective ventilation strategy (4). The respiratory parameters were specified as follows: oxygen concentration was 25%, ventilation frequency was 15 times/min, inspiration and expiration ratio was 1:2, tidal volume was 6 mL/kg, positive end-expiratory pressure was 5 cm H_2_O, inspiratory oxygen concentration and respiratory rate were adjusted accordingly to maintain the oxygen saturation above 95%, and end-expiratory carbon dioxide concentration was 30-40 mmHg. The status of the animals 30 min after surgery was recorded as the reference status, and the timing was recorded as “Ba.” Then the ARDS pig models were prepared by intravenously injecting the animals with 20 μg/kg endotoxin (*Escherichia coli* 055: B5, Sigma, USA) (8 [Bibr B09]). Upon successful modeling, the two groups of animals were given sedative and analgesic, fluid infusion (10-15 mL·kg^-1^·h^-1^), auxiliary breathing machine, heparin anticoagulation, and other treatment. The experimental group was given continuous veno-venous hemofiltration treatment (Jinbao blood filter and M60 membrane, USA). The activated clotting time of the two groups were maintained at about 180-250 s. The parameters of the blood filtration were configured as 50 mL/h for the blood flow, 300 mL/h for the replacement fluid speed, and 20 mL/h for the ultrafiltration.

**Figure 1:**
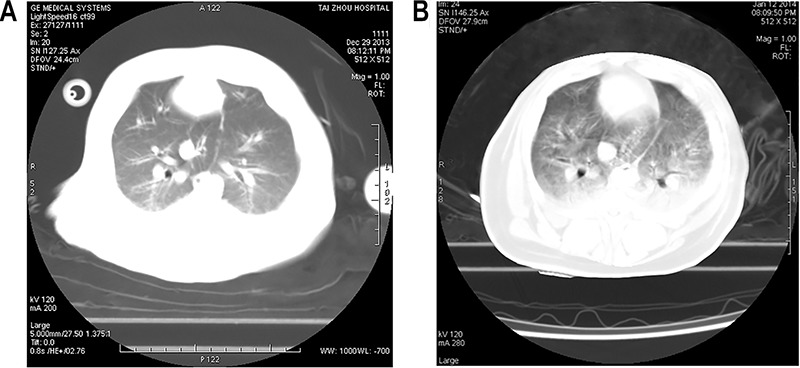
Pulmonary CT test of acute respiratory distress syndrome piglet model. *A*, Before modeling, lungs were normal; *B*, after modeling, lungs showed diffuse infiltrate and pulmonary atelectasis.

*Modeling success criteria*. According to the Berlin definition of ARDS, the hypoxemia is stratified to include mild (PaO_2_/FiO_2_ <300 mmHg), moderate (PaO_2_/FiO_2_ <200 mmHg), and severe (PaO_2_/FiO_2_ <100 mmHg) (2). When the ratio of partial pressure of arterial oxygen (PaO_2_) to fraction of inspired oxygen (FiO_2_) dropped to <300, the CT scan of the lungs of ARDS pig models was performed. The test results indicated that diffuse infiltrative shadows and atelectasis were seen at bilateral lungs (Figure 1). The successful modeling time was recorded as “0 h.”

### Dynamic lung compliance

Dynamic lung compliance (Cdyn) = Tidal volume / (Peak inspiration pressure - Positive end-expiratory pressure).

### Oxygenation index

To conduct blood gas analysis (Abbott Laboratories i-STAT 300, USA), a 5-mL previously heparinized blood syringe was used to draw about 2 mL arterial blood from the preinstalled femoral artery catheter, which was then sealed and isolated from air with a rubber plug.

### Hemodynamics

The variations in heart rate and mean blood pressure were recorded using the PICCO monitor. Water (10 mL) at 4°C was then injected into the internal jugular vein within 10 s. On the basis of the heat release waveform, the cardiac index, pulmonary vascular permeability index, and extravascular lung water index were calculated.

### Determination of levels of tumor necrosis factor α, interleukin 6, and interleukin 10 in serum and BALF

At time points Ba, 0, 2, 4, and 6 h, the bronchoalveolar lavage fluid (BALF) test was conducted following the test procedure elaborated as follows: First, 20 mL saline was injected to both the left and right lung bronchia via a bronchoscope. Then most of the saline was recovered after 5 min. About 5 mL of the recovered liquid was centrifuged at 500 *g* for 5 min at 4°C, and the supernatant was collected, repacked, and stored at −80°C. At time points Ba, 0, 2, 4, and 6 h, 4 µL of blood was taken and centrifuged 500 *g* for 5 min at 4°C, and the supernatant was collected, repacked, and stored at -80°C for further inspection. Finally, it was inspected using an enzyme-linked immunosorbent assay kit (Hermes Criterion Biotechnology, Canada), following the manufacturer’s instructions. The absorbance values of the standard product and the samples were measured using a microplate reader. The standard curve and the corresponding regression equation were depicted, and the corresponding concentration of each sample was calculated.

### Pathological detection

The tissues of the heart, lung, kidney, and other organs were extracted, fixed with formalin, dehydrated and wrapped with paraffin, and stained with hematoxylin-eosin. The pathological changes were then observed under an optical microscope.

### Detection of nuclear factor kappa B p65 protein expression in the heart, lung, and kidney tissues

Fresh lung tissue (100 mg) was frozen in liquid nitrogen and ground into powder. Then, 1 mL pyrolysis liquid was added and the total protein was extracted. Finally, the protein concentration was determined using the bicinchoninic acid assay. After this, 10 µg protein was put through 10% sodium dodecyl sulfate-polyacrylamide gel electrophoresis at 15 V, dried for 35 min until it was half dried, and printed on a polyvinylidene fluoride membrane. The membrane was then sealed in 5% skimmed milk powder for 2 h at room temperature and washed four times with phosphate-buffered saline with Tween 20 (PBST) for 7 min. The membrane was hatched in the liquid (diluted at a ratio of 1:1000) containing nuclear factor kappa B (NF-κB) p65 pig monoclonal antibody (product code: ab72555, Abcam, USA) and stored at 4°C overnight. The membrane was again washed four times with PBST, and hatched in the liquid (diluted at a ratio of 1:1000) containing horseradish peroxidase-labeled immunoglobulin pig antibody for 2 h at 37°C. Once again the membrane was washed four times with PBST. The membrane was then exposed to a film using enhanced chemiluminescence, which was developed for conducting imaging analysis. Finally, Quantity One 4.31 software (USA) was used to measure the gray scale ratio of each NF-κB strip to each glyceraldehyde-3-phosphate dehydrogenase strip of the western blot method.

### Statistical analysis

All data are reported as means±SD. The *t*-test was used to analyze measurement data, and the *χ* ^2^-test was used to analyze enumeration data. Statistical analysis was conducted using the SPSS 16.0 software (SPSS Inc., USA), and plotted in Excel (Microsoft, USA).

## Results

### Dynamic lung compliance and oxygenation index

Upon successful modeling, the dynamic lung compliance and oxygenation index of both groups of animals decreased significantly. After treatment for 6 h, the experimental group showed better dynamic lung compliance and oxygenation index compared with the control group (P=0.002, P<0.05, respectively; Table 1).

**Table:**
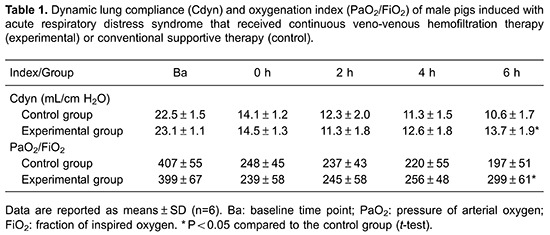

### Hemodynamics

After the endotoxin infusion, both groups experienced major changes in the heart rate, mean arterial blood pressure, cardiac index, pulmonary vascular permeability index, and extravascular lung water index in reference to their baseline values, which are indicated as follows: 1) At time points Ba and 0 h, no significant difference existed between the two groups. At time points 4 and 6 h, the experimental group showed slower heart rate, higher mean arterial blood pressure, and decreased extravascular lung water index compared with the control group, and the differences were statistically significant (P=0.020, 0.018, 0.000 at 4 h and P=0.000, 0.045, 0.038 at 6 h, respectively). 2) Upon successful modeling, cardiac index continuously declined. At 6 h, the experimental group outperformed the control group in cardiac index, and the difference between the two groups was statistically significant (P=0.001). 3) Upon successful modeling, the pulmonary vascular permeability index increased sharply, after which no noticeable change ever occurred; therefore, no significant difference was found (P=0.23). 4) The extravascular lung water indexes of the experimental group at time points 4 and 6 h were less than the control group, and the differences were statistically significant (P=0.015 at 4 h and P=0.003 at 6 h, respectively; Table 2).

**Table:**
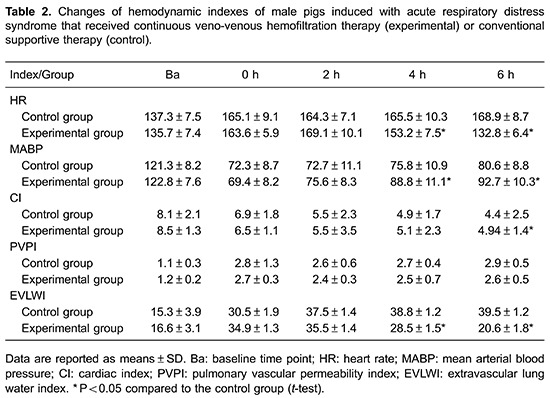

### Urine volume per hour

**Figure 2:**
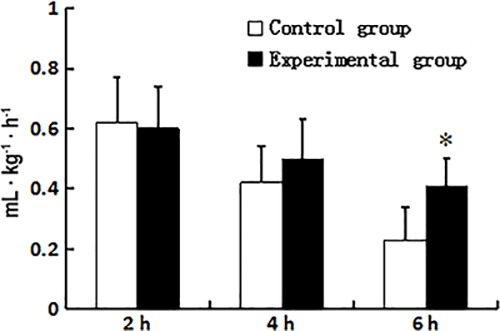
Urine volume per hour in male pigs induced with acute respiratory distress syndrome that received continuous veno-venous hemofiltration therapy (experimental) or conventional supportive therapy (control). The urine volume per hour in the experimental group was significantly higher than in the control group at 6 h (*P<0.05, *t*-test). Data are reported as means±SD.

At time point 6 h, the urine volume per hour of the experimental group was higher than that of the control group, and the difference was statistically significant (P=0.02, Figure 2).

### Changes of inflammatory factors in serum and BALF

The comparison of the inflammatory factors in the serum of the two groups revealed statistically significant differences between the groups at certain time points. Specifically, relative to the control group, tumor necrosis factor-α (TNF-α) at time point 6 h, interleukin 6 (IL-6) at time points 4 and 6 h, and IL-10 at time point 6 h were lower in the experimental group. Compared with the inflammatory factors in the BALF of the control group, TNF-α and IL-6 at time point 6 h were lower with a significant difference of the experimental group, but IL-10 was not statistically different (Tables 3 and 4).

**Table:**
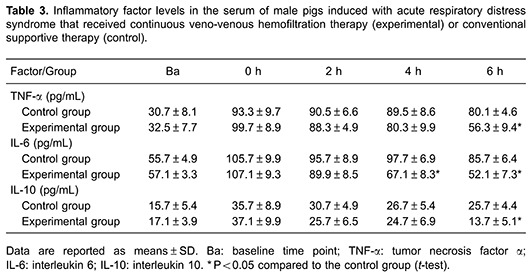

**Table:**
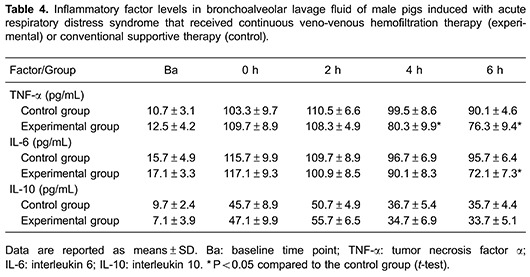

### Histopathological examination

**Figure 3:**
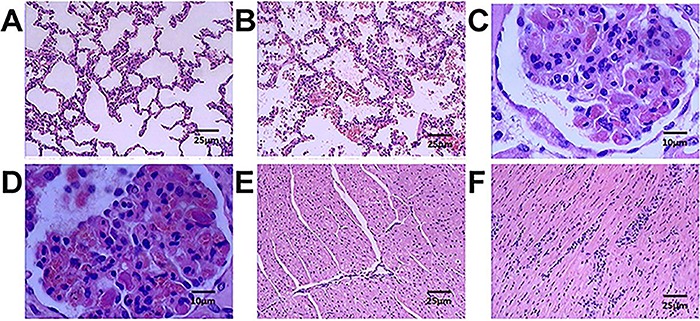
Photographs of pathological findings in tissues from male pigs induced with acute respiratory distress syndrome that received continuous veno-venous hemofiltration therapy (experimental) or conventional supportive therapy (control). *A*, Lung tissue of the experimental group, where slightly thickened alveolar walls are seen, with no obvious leukocyte infiltration. *B*, Lung tissue of the control group, where wider alveolar septa, fibrous tissue hyperplasia, and scattered infiltration in lymphocytes, plasma cells, and neutrophils are seen. *C*, Kidney tissue of the experimental group, where the glomerulus is almost normal. *D*, Kidney tissue of the control group, where a little extravasated blood in glomerulus and foci of necrosis are seen. *E*, Heart tissue of the experimental group, which is essentially normal. *F*, Heart tissue of the control group, where the scattered infiltration of myocardial interstitial is seen in the lymphocytes and plasma cells.

The pathological pictures of the experimental group presented almost a normal structure of the tissues with no obvious infiltration of inflammatory cells (Figure 3A, C, and E). The pathological pictures of the control group presented wider alveolar septa, fibrous tissue hyperplasia, scattered infiltration in lymphocytes, plasma cells, and neutrophils, a little extravasated blood in glomerulus and foci of necrosis, and scattered infiltration of myocardial interstitial in the lymphocytes and plasma cells (Figure 3B, D, and F).

### NF-κB p65 protein content of the heart, lung, and kidney tissues

**Figure 4:**
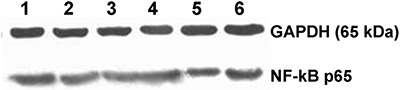
Western blot detection of the NF-κB p65 protein content of the heart, lung, and kidney tissues of male pigs induced with acute respiratory distress syndrome that received continuous veno-venous hemofiltration therapy (experimental) or conventional supportive therapy (control). The numbers 1, 3, and 5 exhibit the NF-κB p65 protein content of the heart, lung, and kidney tissues of the control group, respectively; 2, 4, and 6 exhibit the NF-κB p65 protein content of the heart, lung, and kidney tissues of the experimental group, respectively.

Western blot detection revealed the NF-κB p65 protein content of the tissue extracts (Figure 4). NF-κB p65 protein was found in the heart, lung, and kidney tissues, but the protein content of the experimental group had no significant difference compared with the control group (P<0.05).

## Discussion

ARDS is one of the difficult clinical problems among the intensive care unit patients, which currently does not have efficient drugs and is mainly managed symptomatically (2). Inflammatory disorders probably are the main pathogenesis of sepsis-related ARDS. While CBP can non-selectively remove the molecular inflammation medium of various sizes by diffusion, convection, and adsorption, it provides a new method for treating ARDS.

The present study adopted the ARDS model induced by endotoxin, which simulated well the clinical characteristics of patients with ARDS. Shortness of breath, lower oxygenation index, poorer dynamic lung compliance, significantly decreased mean arterial blood pressure, and significantly increased TNF-α and IL-6 in serum and BALF were observed after an intravenous injection of endotoxin. After CBP treatment, the dynamic lung compliance and oxygenation index of the experimental group improved significantly. These findings were similar to the continuous hemofiltration experiment results obtained by Ullrich et al. (9) using home pigs with endotoxin-induced acute lung injury. These results were also consistent with those obtained by Dicarlo et al. (10) in a continuous hemofiltration experiment conducted on 27 patients who got acute lung injury after bone marrow transplantation operation. They observed a significantly increased oxygenation index and significantly lower alveolar-arterial oxygen pressure difference in patients during the treatment. The clinical significance of the oxygenation index improvement was that the lung function was better because of reduced intrapulmonary shunt and improved V/Q ratio. Thus, CBP treatment obviously improves blood flow dynamics. The reasons could be that it reduces inflammation levels and relieves the inhibition of inflammatory mediators on cardiovascular function; directly clears endogenous substances such as NO and atrial natriuretic peptide that may expand blood vessels or inhibit myocardial systolic functions; adjusts the balance between positive material such as catecholamine and negative material; and improves oxygenation and restores metabolism of cardiovascular system. This study found that CBP treatment removed inflammatory factors such as TNF-α, IL-6, and IL-10 in serum and BALF, and thus nonselectively inhibited the inflammation medium, which was consistent with previous studies (11,12). At present, lung water content level and pulmonary edema are believed to be associated with the survival rate of patients with ARDS (13). This study also found that CBP treatment reduced the lung water content, which was quite useful for treating ARDS. It reduced the alveolar edema and alveolar interstitial edema of lung-injured animals, at the same time it restored the active substance concentration of the alveolar inner surface, alleviated the alveolar collapse, and promoted oxygenation. The pathological analysis of the heart, lung, and kidney tissues revealed that CBP treatment reduced tissue damage. Therefore, besides protecting the lung, it also protected other organs such as heart and kidney. This study also observed that CBP treatment reduced the lung water content and improved urine volume and blood flow dynamics. The pathological analysis revealed that the injury of the experimental group, as well as the inflammation infiltration, were alleviated compared with that of the control group. Although CBP treatment for ARDS patients has not been written in guidelines, its functions such as clearance of inflammatory mediators, suppression of body reaction to pro-inflammation and anti-inflammation, stabilization of hemodynamics, and improvement of organ functions have been reported and adopted many times in the recent decade (9,10,12,14). To summurize, at present, the main mechanisms of blood purification in the treatment of ARDS are ultrafiltration of liquid, maintenance of a low temperature for reduction of oxygen consumption, reduction of extravascular lung water content, and elimination of inflammation factors (15).

This study confirmed that CBP treatment can improve the functions of heart, lung, kidney, and other organs of piglets affected with endotoxin-induced ARDS, and to some extent, protect these organs. Due to its nonselective removal of inflammation, it also clears those mediums that boost the decay of inflammation. For example, this study found that IL-10 was also removed, although the NF-κB p65 protein content of the tissues of heart, lung, and kidney did not decrease after 6 h of CBP treatment. Transcription factor NF-kB, when activated by cytokines or reactive oxygen/nitrogen species, induces iNOS and COX-2. It is also involved in the pathogenesis of acute lung injury (16,17 [Bibr B18]), but an acidic environment and presence of surfactants may reduce NF-kB activation as has already been demonstrated in a previous study (18). This study reported a difference in NF-κB activation and protein content between the experimental group and the control group with a prolonged CBP treatment. Therefore, CBP treatment can remove inflammatory mediators and suppress inflammation reaction, but its long-term effect on boosting inflammation decay is unknown and needs further research. In addition, some studies found that CBP treatment may suppress the inflammation reaction through the Toll/NF-κB signal path, which could not be observed in the present experiment. Whether it was missed owing to the limited hemofiltration time needs further study.

It can be concluded that, under the present experimental conditions, CBP treatment improved the functions of heart, lung, kidney, and other organs of piglets affected with endotoxin-induced ARDS, and to some extent, protected these organs. Its protective mechanism may be related to elimination of inflammation medium, improvement of circulation, alleviation of lung edema, and promotion of oxygenation. CBP treatment, however, cannot reduce the NF-κB p65 content of the tissues.
